# Multidrug-resistant Tuberculosis Management in Resource-limited Settings

**DOI:** 10.3201/eid1209.051618

**Published:** 2006-09

**Authors:** Eva Nathanson, Catharina Lambregts-van Weezenbeek, Michael L. Rich, Rajesh Gupta, Jaime Bayona, Kai Blöndal, José A. Caminero, J. Peter Cegielski, Manfred Danilovits, Marcos A. Espinal, Vahur Hollo, Ernesto Jaramillo, Vaira Leimane, Carole D. Mitnick, Joia S. Mukherjee, Paul Nunn, Alexander Pasechnikov, Thelma Tupasi, Charles Wells, Mario C. Raviglione

**Affiliations:** * World Health Organization, Geneva, Switzerland;; † KNCV Tuberculosis Foundation, The Hague, the Netherlands;; ‡ Partners In Health, Boston, Massachusetts, USA;; § Socios En Salud, Lima, Peru;; ¶ International Union Against Tuberculosis and Lung Disease, Paris, France;; # Centers for Disease Control and Prevention, Atlanta, Georgia, USA;; ** Tartu University Clinics, Tartu, Estonia;; †† National TB Programme, Tallinn, Estonia;; ‡‡ State Centre of Tuberculosis and Lung Diseases of Latvia, Riga, Latvia;; §§ Harvard Medical School, Boston, Massachusetts, USA;; ¶¶ MDR-TB Project in Tomsk Oblast, Tomsk, Russian Federation;; ## Makati Medical Center, Makati, the Philippines

**Keywords:** Tuberculosis, Multidrug-Resistant, Prevention and control, Antitubercular Agents, research

## Abstract

Evidence of successful management of multidrug-resistant tuberculosis (MDRTB) is mainly generated from referral hospitals in high-income countries. We evaluate the management of MDRTB in 5 resource-limited countries: Estonia, Latvia, Peru, the Philippines, and the Russian Federation. All projects were approved by the Green Light Committee for access to quality-assured second-line drugs provided at reduced price for MDRTB management. Of 1,047 MDRTB patients evaluated, 119 (11%) were new, and 928 (89%) had received treatment previously. More than 50% of previously treated patients had received both first- and second-line drugs, and 65% of all patients had infections that were resistant to both first- and second-line drugs. Treatment was successful in 70% of all patients, but success rate was higher among new (77%) than among previously treated patients (69%). In resource-limited settings, treatment of MDRTB provided through, or in collaboration with, national TB programs can yield results similar to those from wealthier settings.

## Abstract 2

Managing MDRTB through national programs can yield results similar to those seen in wealthier settings.

Multidrug-resistant tuberculosis (MDRTB), defined as TB resistant to at least isoniazid and rifampin, represents an obstacle to TB control, especially in areas where MDRTB prevalence is high (*1*). New World Health Organization (WHO) estimates suggest that 424,203 MDRTB cases occurred in 2004 (95% confidence interval 376,019–620,061), or 4.3% of all new and previously treated TB cases. More than half of the estimated MDRTB cases were in China and India, while the highest estimated prevalences were in countries of the former Soviet Union and certain provinces of China (*2*).

DOTS is the internationally recommended strategy for TB control and is based on a 6-month treatment regimen with first-line drugs (isoniazid, rifampin, pyrazinamide, and ethambutol) for new patients and an 8-month treatment regimen with isoniazid, rifampin, pyrazinamide, ethambutol, and streptomycin for re-treatment patients (*3*). While DOTS prevents the emergence of drug resistance in drug-susceptible cases, in patients with MDRTB, this treatment yields inadequate cure rates (*4**–**7*). A retrospective cohort study of treatment of MDRTB with this regimen in 6 countries showed treatment success rates of 52% (range 11%–60%) in new cases and 29% (range 18%–36%) in previously treated cases (*5*). In addition, the frequency of TB recurrence among MDRTB patients previously considered to be cured after this treatment has been reported at 28% (*6*). Treating MDRTB with second-line drugs may cure >65% of patients and stop ongoing transmission (*8**–**10*). However, most of the evidence of successful MDRTB management is generated from high-income countries where treatment is provided in referral hospitals (*10*).

In 1999, WHO and partner agencies launched DOTS-Plus to manage MDRTB in resource-limited settings, a term that was recently abolished since it was used for the piloting of the management of MDRTB within the context of DOTS programs. Effective MDRTB control builds on the 5 tenets of the DOTS strategy (*3*) and expands each of these areas to address the complexities associated with treating MDRTB (*11*). As part of this strategy, a novel partnership known as the Green Light Committee (GLC) was created to foster access to, and rational use of, second-line drugs (*11**–**13*). The second-line drugs included in the WHO Model List of Essential Medicines are amikacin, capreomycin, ciprofloxacin, cycloserine, ethionamide, kanamycin, levofloxacin, ofloxacin, *p*-aminosalicylic acid, and prothionamide (*11*). GLC reviews applications from projects that wish to integrate MDRTB management into a DOTS-based TB control program. If the applicant proposes a strategy consistent with international recommendations and agrees to the monitoring procedures of GLC, then access to reduced-price, quality-assured second-line drugs is granted. Some of the requirements for GLC endorsement include a well-functioning DOTS program, long-term political commitment, rational case-finding strategies, diagnosis of MDRTB through quality-assured culture and drug susceptibility testing (DST), treatment strategies that use second-line drugs under proper management conditions, uninterrupted supply of quality-assured second-line drugs, and a recording and reporting system designed for MDRTB control programs that enables monitoring and evaluation of program performance and treatment outcome (*11**,**13**,**14*). These conditions represent the MDRTB control framework. Projects must be tailored to site-specific epidemiologic and programmatic conditions within this framework. As a result, MDRTB control programs may differ substantially between settings (*11*). Some aspects in which MDRTB control programs may vary include whether all TB patients are tested with culture and DST or only patients with an increased risk for MDRTB, use of standardized or individualized second-line treatment regimen, and hospitalization of MDRTB patients or provision of treatment on an ambulatory basis. This analysis of the first 5 GLC-endorsed MDRTB control programs provides, for the first time, results on management of MDRTB under DOTS-based program conditions in multiple resource-limited countries by using standardized treatment outcome definitions.

## Methods

This is a study of MDRTB patients enrolled in Estonia, Latvia, Lima (Peru), Manila (the Philippines), and Tomsk Oblast (Russian Federation). The data were collected prospectively. The enrollment period started in 1999 for Lima and Manila, 2000 for Latvia and Tomsk, and 2001 for Estonia and ended December 31, 2001. All patients evaluated were managed under GLC-approved protocols and had the opportunity to receive >24 months of treatment. In addition, follow-up data on successfully treated patients were collected at the beginning of 2006, two years after the last patient's treatment ended (December 31, 2003).

A new MDRTB patient was defined as a patient who had never received TB treatment or who had received TB treatment for <1 month. An MDRTB patient previously treated with only first-line drugs was defined as an MDRTB patient who had been treated for >1 month with only first-line anti-TB drugs. An MDRTB patient previously treated with second-line drugs was defined as an MDRTB patient who had been treated for >1 month with >1 second-line anti-TB drug (with or without first-line drugs). Six standard and mutually exclusive categories were used to define treatment outcome: cure, treatment completed, death, default, failure, and transfer out (*14*) (Table 1). The treatment success percentage was obtained by adding the percentage of cured patients to the percentage of patients who completed treatment.

**Table 1:** Treatment outcome definitions for multidrug-resistant tuberculosis (MDRTB) patients (*14*)

Category	Definition
Cure	Completed treatment according to country protocol and been consistently culture-negative (>5 results) for final 12 mo of treatment. If only 1 positive culture is reported during that time with no concomitant clinical evidence of deterioration, patient may still be considered cured, provided that positive culture is followed by >3 consecutive negative cultures taken >30 d apart.
Treatment completed	Completed treatment according to country protocol but does not meet definition for cure or treatment failure because of lack of bacteriologic results (i.e., <5 cultures were performed in the final 12 mo of therapy).
Death	Died for any reason during the course of MDRTB treatment.
Treatment default	Treatment was interrupted for >2 consecutive months for any reason.
Treatment failure	>2 of 5 cultures recorded in the final 12 mo are positive or if any of the final 3 cultures is positive. Treatment will also be considered to have failed if a clinical decision has been made to terminate treatment early due to poor response or adverse events.
Transfer out	Transferred to another reporting and recording unit and the treatment outcome is unknown.

Outcome data were recorded by the individual projects in centralized electronic registers. International standards for core data collection in MDRTB control programs were developed in 2000 (*11*). Projects developed their own standardized forms and electronic databases that included all of the core data elements. Aggregated program and patient data were collected from each project with a data collection form developed by GLC. The accuracy of laboratory methods was verified though regular quality assurance exercises performed by a network of WHO/International Union Against Tuberculosis and Lung Disease supranational TB reference laboratories, as previously described (*1*). For each project, data submitted to WHO were checked for completeness and consistency; all errors or discrepancies were corrected in consultation with the project's investigators.

Statistical tests were performed with the Fisher exact test for 2×2 comparisons and the χ^2^ test for the other tables. For all statistical tests, we regarded a p value <0.05 as significant. Data were analyzed in Stata version 8 (StataCorp LP, College Station, TX, USA).

## Results

The 5 programs are described in Table 2. All projects are conducted in well-established DOTS programs. Four projects are integrated into the national TB program (NTP): Estonia, Latvia, Lima, and Tomsk. The project in Manila is conducted by a nongovernmental organization at a private tertiary hospital, in close collaboration with NTP. All projects provide free care to MDRTB patients. The programs in Estonia, Latvia, and Tomsk are the only available treatment options for MDRTB, while in Lima and Manila, treatment in the private sector is also available.

**Table 2:** Description of MDRTB control programs*

Factor	Estonia	Latvia	Lima	Manila	Tomsk
Start of enrollment	1 Aug 2001	1 Jan 2001	1 Feb 1999	15 Apr 1999	12 Sep 2000
Project size	Country	Country	Region	District	Region
Project population	1,364,101	2,350,000	7,748,258	9,930,000	1,032,400
Prisons included?	Yes	Yes	Yes (1 prison)	No	Yes
% MDRTB, new cases (2002)	11.9	9.8	NA†	NA†	13.7
% MDRTB, previously treated cases (2002)	29.3	26.7	NA†	NA†	43.6
Voluntary HIV counseling/testing?	Yes	Yes	Yes	No	Yes
Empiric regimen?‡	No	Yes	Yes	No	Yes
Surgery used?	Yes	Yes	Yes	No	Yes
DOT (days per wk)	7 hosp, 6 amb	7 hosp, 5–6 amb	6	6	6
Incentives to patients?	Yes	Yes	Yes	No	Yes
Incentives to providers?	Yes	No	Yes	No	Yes
Culture monitoring	Monthly	Monthly	Monthly	Monthly	Monthly
X-ray monitoring	Every 3 mo	Every 3 mo	Every 6 mo	Every 6 mo	Every 3 mo
Drugs for which DST is performed§	H, R, E, S, Z, Amk, Cm, Eth, Km, Ofx, Pth	H, R, E, S, Z, Cm, Cs, Eth, Km, Ofx, Pas, T	H, R, E, S, Z, Amk, Cfx, Cm, Cs, Eth, Km	H, R, E, S, Z, Amc,§ Amk, Km, Cfx, Clr,§ Lfx, Ofx	H, R, E, S, Z, Cm, Cs, Km, Ofx, Pas, Pth

*MDRTB, multidrug-resistant tuberculosis; NA, not applicable; DOT, directly observed treatment; hosp, hospital; amb, ambulatory; DST, drug susceptibility testing; H, isoniazid; R, rifampin; E, ethambutol; S, streptomycin; Z, pyrazinamide; Amk, amikacin; Cm, capreomycin; Eth, ethionamide; Km, kanamycin; Ofx, ofloxacin; Pth, prothionamide; Cs, cycloserine; Pas, p-aminosalicylic acid; T, thiacetazone; Cfx, ciprofloxacin; Amc, amoxicillin–clavulanic acid; Clr, clarithromycin; Lfx, levofloxacin. †Lima and Manila do not perform routine drug resistance surveillance. ‡Use of treatment regimens based on the history of drugs used by the patient while awaiting DST results. §Amc and Clr and are not included on the World Health Organization Model List of Essential Drugs and are therefore not purchased through the Green Light Committee. However, each project used additional drugs at its discretion.

In all projects, financing is obtained through both national healthcare budgets and external sources. All projects work in collaboration with technical agencies and, in Lima and Tomsk, nongovernmental organizations. Directly observed treatment (DOT) is standard care in all projects. Treatment is observed by a range of persons, including healthcare workers (primarily nurses) and community volunteers. DOT worker incentives are provided in Estonia, Lima, and Tomsk, primarily consisting of money (Estonia) and food and money (Lima and Tomsk). Patient incentives, food and free transportation, are provided in all projects except for those in Manila. In Lima, patients also receive housing and social, educational, and financial support, as needed. Lima and Manila offer patient support groups. Sputum culture and DST to first- and second-line drugs are performed at each project site except for Lima and Tomsk, which rely on an international laboratory for DST to second-line drugs. All projects test for susceptibility to several first- and second-line drugs. In 3 projects, those in Estonia, Latvia, and Tomsk, MDRTB patients are hospitalized in a separate ward or building until they are noninfectious. In Peru and Manila, only severely ill patients and patients with side effects are hospitalized.

The 5 projects use different case-finding strategies and treatment options (use of empiric treatment regimens while awaiting DST results or not) (Table 2). In Estonia, Latvia, and Tomsk, all (new and previously treated) patients received DST at the start of treatment. However, in this study MDRTB patients from Tomsk were all previously treated patients on a waiting list for treatment. In Lima, DST is only performed on isolates from patients in whom treatment failed or suspicion of MDRTB is high. Most patients in Lima were referred to the GLC-approved MDRTB control program only after failure of a standardized regimen, which contained second-line drugs and was used by the Peruvian NTP. In Manila, patients had a range of treatment histories; most came after failure of treatment provided by private physicians outside the DOTS program. Because of the long turnaround times for DST results from the international laboratory, patients in Lima and Tomsk often received empiric treatment after culture conversion. For each program, the drugs against which the strains were tested are given in Table 2; however, not all strains were tested against all the drugs listed for each program.

All projects used DST results and previous treatment history to design the individualized regimen. Across the 5 projects, regimens contained >4 drugs, and most patients received >4 drugs initially. All regimens included an injectable agent (amikacin, capreomycin, kanamycin, or streptomycin) and a fluoroquinolone (ciprofloxacin, levofloxacin, or ofloxacin). Nearly all drugs were administered for the duration of treatment except for the injectable agent, which was given for a specified interval after the patient's specimens were culture negative. Treatment duration was 18–24 months, and the exact length was usually determined individually for each patient. The frequency of drugs used in the regimens is shown in Table 3. The median duration of patient follow-up after a patient's having been declared cured or treatment completed was 24 months (range 12 months [Lima and Tomsk] to 36 months [Estonia]).

**Table 3:** Frequency of drugs used in multidrug-resistant tuberculosis control program treatment regimens

Drug	Estonia, n (%)	Latvia, n (%)	Lima, n (%)	Manila, n (%)	Tomsk, n (%)	Total, n (%)
Ethambutol	44 (95.7)	117 (47.8)	102 (20.1)	43 (41.0)	28 (19.6)	334 (31.9)
Pyrazinamide	1 (2.2)	99 (40.4)	146 (28.7)	88 (83.8)	84 (58.7)	418 (39.9)
Streptomycin	1 (2.2)	9 (3.7)	104 (20.5)	51 (48.6)	0	165 (15.8)
Capreomycin	11 (23.9)	115 (46.9)	199 (39.2)	23 (21.9)	94 (65.7)	442 (42.2)
Cycloserine	45 (97.8)	189 (77.1)	316 (62.2)	100 (95.2)	142 (99.3)	792 (75.6)
Ciprofloxacin	0	0	257 (50.6)	18 (17.1)	0	275 (26.3)
Clofazimine	0	0	13 (2.6)	0	0	13 (1.2)
Kanamycin	7 (15.2)	129 (52.7)	167 (32.9)	91 (86.7)	47 (32.9)	441 (42.1)
Levofloxacin	1 (2.2)	0	0	30 (28.6)	0	31 (3.0)
Ofloxacin	35 (76.1)	242 (98.8)	44 (8.7)	87 (82.9)	142 (99.3)	550 (52.5)
p-Aminosalicylic acid	26 (56.5)	71 (29.0)	323 (63.6)	98 (93.3)	118 (82.5)	636 (60.7)
Prothionamide or ethionamide	38 (82.6)	154 (62.9)	244 (48.0)	104 (99.0)	94 (65.7)	634 (60.6)
Augmentin	4 (8.7)	7 (2.9)	325 (64.0)	0	2 (1.4)	338 (32.3)
Clarithromycin	4 (8.7)	1 (0.4)	67 (13.2)	46 (43.8)	3 (2.1)	121 (11.6)
Sparfloxacin	0	0	0	14 (13.3)	0	14 (1.3)
Thiacetazone	0	164 (66.9)	0	0	0	164 (15.7)

Drugs were administered under direct observation. In Lima, Tomsk, and Manila, drugs were administered 6 days per week; in Estonia and Latvia, drugs were given 7 days during the hospital phase and then 5 or 6 days a week after discharge. Monitoring of treatment regimens was based on the results of monthly sputum smear and culture. Chest radiographs were also performed every 3 months in Estonia, Latvia, and Tomsk and every 6 months in Lima and Manila. All projects except that in Manila had access to adjunctive surgery for major interventions such as lung resection. Each project provided patients with ancillary drugs to manage adverse events.

MDRTB program cohort characteristics are shown in Table 4. Among 1,047 MDRTB patients, 119 (11%) were new, and 928 (89%) were previously treated. Among the 919 previously treated patients from whom details could be obtained, 438 (48%) had received only first-line drugs and 481 (52%) first and second-line drugs. Few patients' isolates were resistant to only rifampin and isoniazid (2.6%); most (65%) were resistant to first- and second-line drugs. HIV coinfection was identified in 0% (Estonia and Tomsk) and 4.5% (Latvia) of patients. (In Lima and Tomsk, all MDRTB patients were tested for HIV; in Estonia and Latvia, 67% and 90% of MDRTB patients were tested; and in Manila HIV testing was not performed.) Frequency of hospitalization varied from 5.0% (Manila) to 100% (Latvia), and duration of hospitalization ranged from 29 days (Manila) to 267 days (Tomsk).

**Table 4:** Multidrug-resistant tuberculosis control program cohort characteristics*

Characteristic	Estonia, n (%)	Latvia, n (%)	Lima, n (%)	Manila, n (%)	Tomsk, n (%)	Total, n (%)
Total no. cases	46 (100.0)	245 (100.0)	508 (100.0)	105 (100.0)	143 (100.0)	1,047 (100.0)
New cases	22 (47.8)	91 (37.1)	1 (0.2)	5 (4.8)	0	119 (11.4)
Previously treated cases	24 (52.2)	154 (62.9)	507 (99.8)	100 (95.2)	143 (100.0)	928 (88.6)
Cases previously treated with first-line drugs	19 (79.2)	132 (85.7)	125 (25.0)†	53 (54.6)†	109 (76.2)	438 (47.7)
Cases previously treated with first- and second-line drugs	5 (20.8)	22 (14.3)	376 (75.0)†	44 (45.4)†	34 (23.8)	481 (52.3)
Resistance to only H and R	0	7 (2.9)	11 (2.2)	9 (8.6)	0	27 (2.6)
Resistance to only H, R, and other first-line drugs	0	78 (31.8)	182 (35.8)	26 (24.8)	55 (38.5)	341 (32.6)
Resistance to first- and second-line drugs	46 (100.0)	160 (65.3)	315 (62.0)	70 (66.7)	88 (61.5)	679 (64.9)
Treatment cessation because of adverse events	3 (6.5)	5 (2.0)	NA	9 (8.6)	0	17 (3.2)
HIV coinfection	0	11 (4.5)	5 (1.0)	NA	0	16 (1.7)
Surgery performed	1 (2.2)	18 (7.3)	78 (15.4)	0	17 (11.9)	114 (10.9)
No. patients hospitalized	41 (89.1)	245 (100.0)	NA	5 (5.0)	71 (49.7)	362 (67.2)
Average no. drugs in treatment regimen	5.4	5.5	NA	6.28	5.3	

*H, isoniazid; R, rifampin; NA, not applicable. †Information is lacking from 6 patients (Lima) and 3 patients (Manila) on previous treatment with first-line drugs only or with first- and second-line drugs.

**Figure 1:**
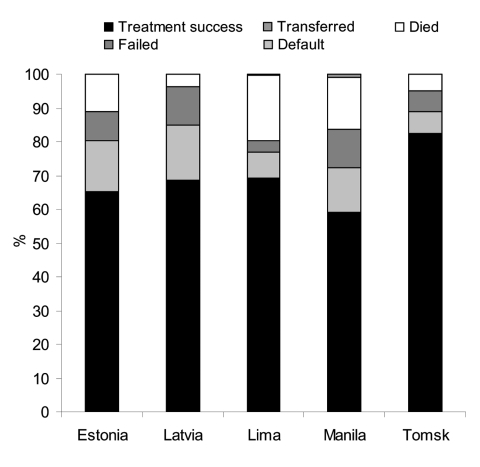
Treatment outcomes of multidrug-resistant tuberculosis patients in Estonia (46 patients), Latvia (245 patients), Lima (508 patients), Manila (105 patients), and Tomsk (143 patients).

The treatment outcomes of new, previously treated, and all MDRTB patients are shown in Table 5 and Figure 1. Treatment was successful in 70% of 1,047 patients (range 59%–83%). Failure occurred in 3.3% to 11% of patients, default in 6.3% to 16%, and death in 3.7% to 19%. In Estonia and Latvia, MDRTB patients not previously treated for TB had a higher treatment success rate (80% vs. 61%, odds ratio [OR] 2.54, 95% confidence interval [CI] 1.47–4.37, p<0.005) and a lower failure rate (4.4% vs. 15%, OR 0.26, 95% CI 0.10–0.67, p<0.005) than previously treated patients. Adverse events led to treatment cessation in 3.2% of patients (range 0% [Tomsk] to 8.6% [Manila]). By the end of 2005, a total of 14 of 670 patients (2.1%) who were followed-up after cure or treatment completion had relapsed (range 1.1% [Lima] to 10.0% [Estonia]) (Table 6).

**Table 5:** Treatment outcomes of new, previously treated, and all multidrug-resistant tuberculosis patients

Patients	Estonia, n (%)	Latvia, n (%)	Lima, n (%)	Manila, n (%)	Tomsk, n (%)	Total, n (%)
New patients						
Cured	16 (72.7)	72 (79.1)	0	1 (20.0)	0	89 (74.8)
Completed	1 (4.5)	1 (1.1)	0	1 (20.0)	0	3 (2.5)
Default	3 (13.6)	13 (14.3)	0	2 (40.0)	0	18 (15.1)
Failed	1 (4.5)	4 (4.4)	0	0	0	5 (4.2)
Died	1 (4.5)	1 (1.1)	1 (100.0)	1 (20.0)	0	4 (3.4)
Transferred	0	0	0	0	0	0
Total	22 (100.0)	91 (100.0)	1 (100.0)	5 (100.0)	0	119 (100.0)
Treatment success	17 (77.3)	73 (80.2)	0	2 (40.0)	0	92 (77.3)
Previously treated patients						
Cured	12 (50.0)	93 (60.4)	351 (69.2)	60 (60.0)	118 (82.5)	634 (68.3)
Completed	1 (4.2)	2 (1.3)	0	0	0	3 (0.3)
Default	4 (16.7)	27 (17.5)	40 (7.9)	12 (12.0)	9 (6.3)	92 (9.9)
Failed	3 (12.5)	24 (15.6)	17 (3.4)	12 (12.0)	9 (6.3)	65 (7.0)
Died	4 (16.7)	8 (5.2)	98 (19.3)	15 (15.0)	7 (4.9)	132 (14.2)
Transferred	0	0	1 (0.2)	1 (1.0)	0	2 (0.2)
Total	24 (100.0)	154 (100.0)	507 (100.0)	100 (100.0)	143 (100.0)	928 (100.0)
Treatment success	13 (54.2)	95 (61.7)	351 (69.2)	60 (60.0)	118 (82.5)	637 (68.6)
All patients						
Cured	28 (60.9)	165 (67.3)	351 (69.1)	61 (58.2)	118 (82.5)	723 (69.1)
Completed	2 (4.3)	3 (1.2)	0	1 (1.0)	0	6 (0.6)
Default	7 (15.2)	40 (16.3)	40 (7.9)	14 (13.3)	9 (6.3)	110 (10.5)
Failed	4 (8.6)	28 (11.4)	17 (3.3)	12 (11.4)	9 (6.3)	70 (6.7)
Died	5 (10.9)	9 (3.7)	99 (19.5)	16 (15.2)	7 (4.9)	136 (13.0)
Transferred	0	0	1 (0.2)	1 (1.0)	0	2 (0.2)
Total	46 (100.0)	245 (100.0)	508 (100.0)	105 (100.0)	143 (100.0)	1047 (100.0)
Treatment success	30 (65.2)	168 (68.6)	351 (69.1)	62 (59.0)	118 (82.5)	729 (69.6)

**Table 6:** Clinical and bacteriologic progress after cure or treatment completion and number of relapses identified by the beginning of 2006

Characteristic	Estonia	Latvia	Lima	Manila	Tomsk	Total
Duration of follow-up after cure or treatment completion (mo)	36	24	12	24	12	
Frequency of follow-up after cure or treatment completion	Every 6 mo	Every 6 mo	Every 3 mo	Every 6 mo	Every 6 mo	
No. cured or completed treatment	30	168	351	62	118	729
No. followed up*	30	168	351	62	59	670
No. cured or completed treatment who relapsed by 2006	3	5	4	1	1	14
Relapse rate (95% confidence interval)	10.0% (2.11–26.53)	3.0% (0.97–6.81)	1.1% (0.31–2.89)	1.6% (0.04–8.66)	1.7% (0.04–9.09)	2.1% (1.14–3.51)

*In Tomsk, 59 patients left the region after having been declared cured. These patients were lost to follow-up.

## Discussion

Today, management of MDRTB is included as a recommended part of the new Stop TB Strategy (*15*). WHO's guidelines have also been revised to encourage countries to collect drug resistance surveillance data from patients in different retreatment categories and to build capacity to diagnose and treat MDRTB within the context of DOTS (*16*). However, few NTPs in resource-limited settings have integrated effective treatment strategies for resistant cases (*17*).

The major perceived barrier to MDRTB treatment is the high cost of quality-assured second-line drugs. Additional barriers include extensive laboratory and monitoring requirements, adverse events associated with second-line drugs, low availability of quality-assured second-line drugs, difficulties in ensuring adequate patient support (including DOT) during the long treatment course, and the risk for resistance to second-line drugs (*18**,**19*). Consequently, many NTPs focus on achieving high cure rates in their DOTS programs and neither diagnose nor treat MDRTB (*17*).

This study represents the first multicountry evaluation of MDRTB patients treated in resource-limited settings under the GLC mechanism and endorsed by the respective NTP of each country. Although program design and patient management varied, the results show that treating MDRTB in resource-limited settings is feasible and effective. Treatment with second-line drugs is more successful than a 6- to 8-month regimen of first-line drugs for such patients and, in spite of a patient population characterized by high proportions of severe chronic cases with extensive resistance patterns, treatment outcomes of these projects match the outcomes of treatment with second-line drugs in wealthier settings (*10*). However, in each project, extensive training on managerial, laboratory, clinical, and social aspects of MDRTB control took place before GLC approval and initiation of treatment. Socioeconomic support was provided to the patients in 4 of the 5 sites, and in all sites a patient-centered approach was used for treatment delivery, with DOT ensured during the full course of treatment. These efforts may partly explain why the relapse rates were low (2.1%) and suggest such best practices are essential for a successful outcome. In addition, all projects were supported by technical agencies, and some benefited from extensive NGO support.

Significant differences were seen in favorable (cure and completed) and unfavorable (default, failure, died, and transferred out) outcomes between projects (p = 0.002), and although patient populations cannot be compared between projects as a result of different TB epidemiologic features in different countries, some general observations can be made with respect to the differences in treatment outcomes. Default rates were higher in Estonia, Latvia, and Manila than in Lima and Tomsk. TB specialists in Estonia and Latvia attributed the high default rates to a high proportion of patients with severe alcohol abuse disorders for whom adherence to treatment is difficult. A recent study in Latvia could not confirm that alcohol misuse was clearly linked to default, but the number of nonadherent patients was small and the statistical power correspondingly weak (*9*). Although the project in Tomsk also experienced problems with alcoholism, default rates were low because a large proportion of patients were imprisoned (41%) during the treatment period. The high default rate in Manila appeared to be related to the facts that at the beginning of Manila's project, treatment was delivered in a single site that was not easily accessible to all patients and that drugs to manage adverse reactions had to be purchased by the patients. In addition, during the reporting period, the program in Manila did not provide any patient or DOT worker incentives. The low default rates in Lima and Tomsk could be attributed to a large variety of treatment delivery options and incentive and enabler programs for patients.

**Figure 2:**
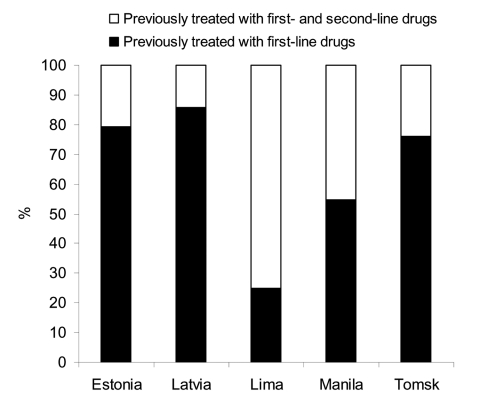
Proportion of multidrug-resistant tuberculosis patients in the 5 sites previously treated with first-line drugs only or with first- and second-line drugs.

The high frequency of death in Lima likely reflects the fact that in a high proportion of patients, a standard MDRTB treatment regimen with second-line drugs was unsuccessful (*20*). The proportion of patients previously treated with second-line drugs was much higher in Lima (75%) than in other projects (14%–45%) (Figure 2). However, the proportions of patients with infections resistant to first- and second-line drugs were similar in Latvia, Lima, Manila, and Tomsk (p = 0.47). In Estonia, resistance patterns to first- and second-line drugs differed substantially when compared with patterns in the other 4 projects (p<0.0001), and in Estonia all patients had infections resistant to first- and second-line drugs (Table 4).

During the study period, only Estonia and Latvia routinely attempted to identify MDRTB patients at the start of their first treatment for TB, and the results show that early identification and referral may reduce death and treatment failure and thus improve treatment success. This finding is consistent with those of Turett et al. (*21*). The delay in the diagnosis of MDRTB results in treatment of patients with chronic disease, progressive parenchymal destruction, higher bacillary loads, and continuing transmission (*22*).

The study confirms that adverse events are manageable in the treatment of MDRTB in resource-limited settings. Few patients stopped treatment because of adverse events, which is similar to a previous report. Each project, however, applied intensive approaches to manage adverse events, including altering dosages when appropriate, administering ancillary drugs to treat adverse events, and discontinuing drugs. In addition, all projects conducted special training on adverse events to second-line drugs and used standard protocols for their registration (*23*).

Studies of the cost and cost-effectiveness of MDRTB management have been completed in Estonia (unpub. data), Manila (*24*), and Tomsk (*25*). From the health system perspective, the average cost per patient treated was approximately US $3,400 in the Philippines and US $9,000–$10,000 in Estonia and Tomsk. The higher costs in Estonia and Tomsk reflect considerable hospitalization during treatment (30%–50% of overall costs compared to 3% in the Philippines). The second-line drug costs ranged from US $1,600 in the Philippines to US $3,700 in Tomsk; second-line drugs were the highest cost items in the Philippines and Tomsk and the second highest in Estonia.

Our study has several limitations. First, risk factors for poor treatment outcomes could not be examined because data were in an aggregate form, not as individual patient data. The second limitation is that the results are not representative of all GLC-approved projects currently functioning. As mentioned, GLC projects are tailored to the local health infrastructure, human and financial resources, and the epidemiologic situation. As a result, costs and outcomes differ between projects. Several projects have been approved by GLC that use standardized treatment regimens based on representative drug resistance surveillance data in relevant patient categories. In settings without a history of second-line drug use, MDRTB control is likely to yield better treatment outcome results. In these settings, susceptibility to the most effective second-line drugs may be preserved, permitting perhaps shorter regimens with fewer, less toxic drugs. As all GLC-approved MDRTB control projects record the same core data, information on success within each of the different approaches will be available within the next 3 years.

## Conclusion

After successful piloting of MDRTB management within TB control programs, WHO and partners have reached the phase of expanding MDRTB control as a component of a comprehensive TB control program, which is described in the WHO guidelines for the treatment of TB (*3*), the new Stop TB Strategy (*15*), and in the new WHO guidelines for the programmatic management of drug-resistant tuberculosis (*26*). As countries are purchasing and using second-line drugs, the likelihood of misuse and creation of strains of TB resistant to all known anti-TB drugs increases. The GLC mechanism offers a way to provide access to care while ensuring rational and effective use of drugs. Beginning in 2002, the Global Fund to Fight AIDS, Tuberculosis and Malaria (GFATM) mandated that requests for second-line drugs for managing MDRTB should go through GLC to prevent their misuse. The GLC model has been proposed to improve access to malaria (*27*) and HIV/AIDS treatment (*28**,**29*). As of May 2006, a total of 41 MDRTB control projects in 37 countries were endorsed by GLC, and >21,000 MDRTB patients were approved for treatment. The number of GLC-approved MDRTB control programs is increasing rapidly, both as a result of more funding for TB control from the GFATM and mainstreaming of MDRTB management into general TB control efforts. However, with the estimated incidence of 424,203 MDRTB cases, most cases remain undiagnosed and untreated. Expanding projects and accelerating evidence gathering are necessary to further develop international policies. The future success of MDRTB management in resource-limited settings will depend on the ability of the donor community and technical agencies, as well as TB-endemic countries themselves, to expand and strengthen MDRTB control programs.
